# Genomic and clinical predictors of cardiovascular disease in Familial dyslipidemia: risk stratification in Egyptian adolescents and young adults

**DOI:** 10.1186/s12944-025-02814-0

**Published:** 2025-12-15

**Authors:** Ammal M. Metwally, Nesma M. Elaraby, Wafaa M. Ezzat, Mark O. Dimitry, Ghada A. Elshaarawy, Neveen A. Ashaat, Ashraf Reda, Ahmed Bendary, Mohamed H. Abbas, Tarek R. El Mawardy, Walaa A. Basha, Iman H. Kamel, Engy A. Ashaat

**Affiliations:** 1 Community Medicine Research Department, Medical Research and Clinical Studies Institute, National Research Centre (Affiliation ID: 60014618), Dokki, Cairo Egypt; 2 Medical Molecular Genetics Department, Human Genetics and Genome Research Institute, National Research Centre, Cairo, Egypt; 3 Internal Medicine Department, Medical Research and Clinical Studies Institute, National Research Centre (Affiliation ID: 60014618), Dokki, Cairo Egypt; 4 Genetics and Biotechnology Department, Ain Shams University, Cairo, Egypt; 5 Cardiology Department, Menofia University, Menofia Governorate, Egypt; 6 Cardiology Department, Faculty of Medicine, Benha University, Qalyubia Governorate, Egypt; 7 Biological Anthropology Department/Medical Research and Clinical Studies Institute, National Research Centre (Affiliation ID: 60014618), Dokki, Cairo, Egypt; 8 Child Health Department, Medical Research and Clinical Studies Institute, National Research Centre (Affiliation ID: 60014618), Dokki, Cairo Egypt; 9 Clinical Genetics Department, Human Genetics and Genome Research Institute, National Research Centre, Cairo, Egypt; 10 Medical Research and Clinical Studies Institute, National Research Centre, P.O. 12622, Dokki, Giza, 60014618 Egypt

**Keywords:** Familial dyslipidemia, Cardiovascular risk prediction, Next-Generation sequencing, LDLR variants, Composite risk score

## Abstract

Familial dyslipidemia (FD), particularly familial hypercholesterolemia (FH), is a major contributor to premature cardiovascular disease (CVD), especially in regions with high consanguinity and underutilized genetic screening, such as Egypt. This study aimed to assess clinical, biochemical, and genetic factors that differentiate FD patients with and without CVD, and to develop a composite risk score for individualized stratification. A cross-sectional study was conducted on 60 Egyptian patients aged 15–25 years with genetically confirmed FD, equally divided based on CVD status. All participants underwent detailed clinical assessment, lipid profiling, and targeted next-generation sequencing of *LDLR*, *APOB*, and *PCSK9* genes. Missense variants were evaluated using SIFT, PolyPhen-2, CADD, and ΔΔG stability scores, and classified according to ACMG criteria. Compared to non-CVD patients, those with CVD had significantly higher triglyceride levels (median: 356.5 vs. 236.5 mg/dL; *p* < 0.001) and a higher frequency of heterozygous pathogenic *LDLR* variants (30.0% vs. 3.3%; *p* = 0.006), while homozygous variants were more common in non-CVD patients (26.7% vs. 0%; *p* = 0.002). Deleterious missense variants were notably more frequent in the CVD group (56.7% vs. 10.0%; *p* < 0.001). A 10-variable composite risk score integrating clinical, lipid, and bioinformatic predictors effectively distinguished high- and moderate-risk cases (AUC = 0.742; *p* = 0.022), with 89.5% sensitivity and 81.8% negative predictive value. The study highlights the importance of combining clinical and genomic data for early risk stratification and introduces a pragmatic tool for identifying high-risk youth in resource-limited, consanguineous populations.

**Supplementary Information:**

The online version contains supplementary material available at 10.1186/s12944-025-02814-0.

## Introduction

 Familial dyslipidemia (FD), particularly FH, is one of the most common inherited metabolic disorders, affecting approximately 1 in 313 individuals globally [1]. Despite this high prevalence, over 90% of cases remain undiagnosed, placing individuals at increased risk of premature coronary artery disease (CAD); up to 20-fold higher than in the general population if untreated [1, 2]. Early intervention with statins, ezetimibe, or *PCSK9* inhibitors has been shown to normalize this risk, particularly when initiated before vascular complications develop [3].

The diagnostic landscape is shifting towards early identification through universal cholesterol screening in children and cascade testing in families [4]. Genetic analysis now plays a central role in confirming FH diagnoses and informing clinical management, especially through identification of pathogenic variants in *LDLR*, *APOB*, and *PCSK9* genes [5, 6]. However, conventional risk tools like the Framingham score often underestimate risk in FH patients, resulting in suboptimal treatment allocation [7, 8]. This has prompted the development of integrative approaches that combine clinical, biochemical, and genomic data for more accurate risk stratification.

In the Eastern Mediterranean Region (EMRO), cardiovascular disease (CVD) contributes to more than 50% of deaths due to noncommunicable diseases [9]. Despite a high burden of hyperlipidemia, familial forms such as FH remain vastly underdiagnosed, particularly in high-consanguinity settings like the Middle East, where the prevalence of homozygous FH is increased [10]. The absence of national registries, limited access to genetic diagnostics, and underutilization of cholesterol screening hinder timely diagnosis. Advanced testing remains largely confined to tertiary care centers in some countries, including Saudi Arabia [11] and Egypt [12].

In Egypt, approximately 46% of all deaths are attributed to CVD [13]. Although national estimates of FH prevalence are lacking, indirect evidence suggests a significant burden, compounded by low screening uptake. Fewer than 9% of Egyptians report ever having their cholesterol measured [14–16]. Genetic testing is rarely available outside of academic research settings or private-sector services [17, 18]. High out-of-pocket expenditures, representing over 60% of total healthcare spending, further restrict access to advanced lipid testing and therapies [19]. Nonetheless, recent national guidelines have prioritized early detection and risk-based treatment, offering a foundation for improved care [20]. Egypt’s robust medical workforce and specialized cardiac centers present an opportunity to implement targeted FH interventions on a broader scale [21].

Risk stratification in FD requires a multidimensional approach that incorporates both genetic and clinical risk factors, especially given the prevalence of comorbidities such as obesity, diabetes, and smoking in the Egyptian population [9]. Early-onset CVD frequently affects individuals during their most economically productive years, with long-term consequences on household income, employment, and health system costs [22, 23]. Yet preventive care remains underutilized, with many individuals facing structural and behavioral barriers to routine checkups and chronic disease management [24, 25].

To address these gaps and enhance cardiovascular risk assessment in FD, this study was designed around three key objectives. First, we aimed to compare the clinical and anthropometric characteristics of FD patients with and without CVD to identify phenotype-based risk indicators. Second, we sought to characterize the spectrum of genetic variants in both groups and assess their predicted functional impact using in silico bioinformatics tools. Finally, we developed a composite risk scoring model that integrates genetic, clinical, and biochemical variables to improve precision in CVD risk prediction among FD patients.

To our knowledge, this is the first Egyptian study to apply an integrated framework combining targeted next-generation sequencing, clinical profiling, and bioinformatics-based functional annotation to compare FD patients with and without cardiovascular disease [26]. Unlike prior research focused mainly on diagnostic prevalence or lipid pattern characterization, this study leverages computational prediction tools (e.g., SIFT, PolyPhen-2, CADD) and structural modeling to interpret variant pathogenicity as an approach not previously applied in this population [27–29].

By establishing a population-specific composite risk score, this study contributes a scalable and context-sensitive model to support clinical decision-making and prioritize high-risk individuals for intensive lipid-lowering interventions. Findings may also inform national policy on cascade screening and personalized CVD prevention strategies tailored to Egypt’s unique genetic and health system landscape.

## Methodology

### Study design and setting

This study employed a cross-sectional comparative design, allowing for the assessment of genomic variants and clinical outcomes (presence or absence of CVD) at a single time point [30]. Participants were recruited from the Congenital Heart Disease and Multiple Congenital Anomalies (CHD & MCA) Clinic and the Internal Medicine Clinic of the Medical Research Centre of Excellence at the National Research Centre (NRC), Cairo, Egypt. Additional referrals were received from cardiac outpatient clinics at Ain Shams, Cairo, Benha, and Menoufia University hospitals, all serving adolescent and young adult dyslipidemia patients. This strategic recruitment ensured access to FD cases, given the low global prevalence of familial hypercholesterolemia, approximately 1 in 313 individuals worldwide [31]. By targeting tertiary care clinics and leveraging a national research network, we obtained a diverse sample of Egyptian FD patients, enhancing the generalizability of the findings within this population.

### Target groups, inclusion and exclusion criteria

The study aimed to identify young individuals with FD, particularly those with monogenic causes, by focusing on patients aged 15–25 years. This age range represents the early-onset phase of FD, where genetic factors play a dominant role before long-standing CVD or other comorbidities develop. Focusing on younger patients enhances the likelihood of detecting causal mutations and early biomarkers, as treatments and advanced atherosclerosis have not yet altered the phenotype significantly.

Participants were included if they met the clinical criteria for FD and were stratified into two groups based on CVD status: Group 1 (FD + CVD) included FD patients with established CVD, and Group 2 (FD without CVD) included FD patients without clinical CVD. All patients met the Simon Broome criteria for FH, ensuring a consistent phenotypic definition of FD. This included an LDL cholesterol level ≥ 4.9 mmol/L (190 mg/dL) in adults (≥ 4.0 mmol/L in those < 16 years), along with either tendon xanthomas in the patient or first/second-degree relatives, or a family history of premature coronary heart disease [32]. Only those meeting the Simon Broome definition of “definite” or “probable” FH were included, ensuring significant hypercholesterolemia accompanied by clinical signs or a family history indicative of a hereditary lipid disorder [33].

Exclusion criteria were applied to avoid confounding factors and ensure focus on primary genetic dyslipidemia. Patients with secondary causes of dyslipidemia, such as uncontrolled diabetes, hypothyroidism, nephrotic syndrome, chronic kidney or liver disease, or those on medications known to affect lipid levels (e.g., corticosteroids, isotretinoin), were excluded to ensure that hyperlipidemia was familial in origin. Additionally, patients with non-dyslipidemic inherited cardiac conditions were excluded to maintain a homogeneous cohort. This selective inclusion enriched the sample for true familial (likely monogenic) dyslipidemia, increasing the study’s power to detect genotype–phenotype correlations. All patients had no contraindications to phlebotomy and were willing to undergo genetic testing.

Furthermore, inclusion criteria required that 30 patients diagnosed with FD had documented CVD, defined as the presence of CAD confirmed by angiography or stress testing, or a history of acute myocardial infarction (AMI), prior coronary revascularization, or other clinical manifestations of atherosclerotic disease. Exclusion criteria included individuals with non-atherosclerotic cardiovascular diseases, such as congenital heart disease or myocarditis, and patients with conditions that could interfere with CVD diagnosis or progression, such as severe renal disease or active inflammatory conditions. Individuals unable to comply with the study protocol or follow-up requirements were also excluded.

### Sample size and sampling technique

Given the rarity of FD in the general population, a purposive sampling strategy was utilized. Instead of random sampling, we deliberately recruited from lipid clinics as discussed before to obtain “information-rich” cases typical of FD [34]. The initial target sample size was 60 index cases (Individuals with FD), with the expectation that this number would provide a preliminary comparison between those with and without CVD. All cases’ first-degree relatives were also approached for cascade screening. All patients were selected for next-generation sequencing (NGS). These 60 cases were chosen to include those with as well as without CVD for comparison (30 cases per group). The sample size of 60 total patients was deemed sufficient to observe large effect-size differences between groups and to uncover prevalent pathogenic variants in this population.

Power calculation was performed due to the exploratory nature of the study and is estimated to be 80% according to the following basis: Group sample sizes of 30 in group one and 30 in group two achieve 80% power to detect a difference between the group proportions of −0.3300. The proportion in group FD with CVD is assumed to be 0.17 [15]. The proportion in group two (due to unknown prevalence) is assumed to be 0.50. The test statistic used is the two-sided Z test with pooled variance. The significance level of the test was targeted at 0.05. The significance level actually achieved by this design is 0.05. The equation was as follows: [35, 36].

### Data collection tools

#### Clinical and anthropometric assessment

Each patient underwent a thorough clinical evaluation and physical examination. A structured questionnaire and medical chart review were used to document demographics, medical history, medication use, and family history of dyslipidemia or premature CVD (e.g. myocardial infarction in male relatives < 55 or female relatives < 65 years). A pedigree was constructed for each case to assess inheritance patterns and to identify additional family members at risk. Anthropometric measurements were obtained with participants wearing light clothing and no shoes. Weight was measured to the nearest 0.1 kg using a calibrated digital scale, and height to the nearest 0.5 cm using a wall-mounted stadiometer. Body mass index (BMI) was then calculated as weight (kg) divided by height (m²). Waist circumference was measured at the midpoint between the lowest rib and iliac crest. Blood pressure was recorded in the right arm after 5 min of rest (three readings averaged). The presence of xanthomas was assessed by a clinician (inspection of Achilles tendons, extensor tendons of hands, elbows, knees, and plantar aspect of feet), and corneal arcus or xanthelasma were noted if present. These clinical findings were important, as features like tendon xanthomas are part of FH diagnostic criteria and indicate a high likelihood of an underlying LDL receptor mutation [32]. All clinical data were collected by trained physicians using a standardized form to ensure consistency.

### Biochemical lipid profiling

Fasting venous blood samples were collected after a 12–14 h overnight fast. Serum was separated immediately and either analyzed or stored at − 80 °C. Each participant underwent a comprehensive lipid profile, including total cholesterol (TC), triglycerides (TG), high-density lipoprotein cholesterol (HDL-C), and low-density lipoprotein cholesterol (LDL-C). TC, TG, and HDL-C levels were measured using enzymatic colorimetric assays on an automated clinical chemistry analyzer, following manufacturer instructions and CDC calibration standards. LDL-C was calculated using the Friedewald formula when TG levels were < 400 mg/dL, or directly measured by immunoassay for higher TG levels. Apolipoprotein B (apoB) was also measured via immunoturbidimetric assay.

To ensure accuracy, two levels of control sera accompanied each assay batch, and the laboratory participated in an external quality assurance program. Standard clinical laboratory protocols were followed to meet international guidelines (e.g., coefficient of variation < 5% for TC and HDL-C assays). Basic metabolic and thyroid function tests were performed to rule out secondary causes of dyslipidemia (e.g., elevated TSH for hypothyroidism). The biochemical data facilitated classification of patients’ lipid abnormalities (e.g., pure hypercholesterolemia vs. combined dyslipidemia).

### Genomic DNA extraction

For the 60 patients selected for genetic analysis, genomic DNA was extracted from peripheral blood leukocytes using standard salting out method. DNA samples of patients were quantified using a NanoDrop 1000 spectrophotometer (Thermo Fisher Scientific, Inc., Waltham, MA). and diluted to a starting concentration of 3.0 ± 0.5 ng/µl and measured using fluorometric Denovix Qubit™ dsDNA BR Assay Kit (ThermoFisher, Waltham, MA, USA). All samples were anonymized using study codes to maintain blinding during genetic analysis.

### Targeted NGS gene panel design

A customized Next-Generation Sequencing (NGS) panel was developed to capture key genes involved in familial dyslipidemia. It targeted all exons and exon–intron boundaries (± 10 bp) of primary FH genes including *LDLR*,* APOB*, and *PCSK9*, which account for most monogenic FH cases [37], along with *LDLRAP1* and other relevant genes such as *APOA5*,* LPL*,* APOC2*,* APOE*,* ANGPTL3*, and *LIPA* to detect other familial dyslipidemia phenotypes. ClinVar and HGMD databases informed panel design.

Target enrichment was performed using Agilent SureSelect kits, and sequencing was carried out on MiSeq sequencer platform (Illumina) achieving 2 × 150 bp paired-end reads with > 100× average coverage per target. Positive control samples with known *LDLR* mutations were included in sequencing runs to validate performance. Sequencing data (FASTQ files) proceeded to bioinformatics processing.

### Bioinformatics and variant calling

The bioinformatics pipeline followed GATK Best Practices. Sequence reads were aligned to the GRCh37/hg19 reference genome using BWA-MEM, duplicates were marked, and local realignment and base recalibration were performed. Variant calling used GATK HaplotypeCaller to generate GVCF files, followed by joint genotyping. Variants were filtered by thresholds (e.g., depth < 20×, Phred score < 30, strand bias) to retain only high-quality calls. Annotation was performed using Ensembl VEP and SnpEff, with additional checks against ClinVar, gnomAD, and LOVD databases.

Copy number variants (CNVs) in *LDLR* were not directly detected by sequencing read depth in this panel; detection was deferred or inferred by family segregation analysis if necessary. Sequencing sensitivity was high, with > 99% of targets covered ≥ 20× and complete identification of known control variants.

### In Silico functional analysis

Nonsynonymous variants were further evaluated using multiple computational prediction tools. SIFT assessed evolutionary conservation, where a score < 0.05 predicted a damaging effect [38]. PolyPhen-2 combined sequence and structural information to categorize variants as benign, possibly damaging, or probably damaging [39]. Mutation Taster used integrated algorithms to predict disease-causing potential [40], and PROVEAN evaluated sequence homology to identify deleterious substitutions (academic.oup.com). Additionally, Combined Annotation Dependent Depletion (CADD) scores were calculated [41], with Phred-scaled scores ≥ 20 indicating high deleteriousness.

The protein folding stability score (ΔΔG) was calculated using I-Mutant 3.0 to estimate the change in Gibbs free energy caused by amino acid substitutions. A ΔΔG value ≤ − 1.5 kcal/mol indicates protein destabilization and was scored as 1; otherwise, it was scored as 0 [42, 43].

When relevant, LDLR crystal structures or homology models were consulted to interpret potential disruption to ligand-binding domains or conserved residues. Concordant predictions across multiple tools were used to increase confidence in variant pathogenicity classification.

### Variant interpretation and classification

All variants were classified according to the American College of Medical Genetics and Genomics/Association for Molecular Pathology (ACMG/AMP) 2015 guidelines [44]. Variants were categorized as pathogenic, likely pathogenic, of uncertain significance (VUS), likely benign, or benign based on population frequency, computational predictions, family co-segregation, functional studies, and ClinVar/ClinGen expert entries.

The ClinGen Familial Hypercholesterolemia Variant Curation Expert Panel refinements (2018–2022) were specifically applied for *LDLR* variants to improve consistency [45]. Two independent clinical geneticists reviewed each variant, and disagreements were resolved through discussion. For example, a novel *LDLR* missense mutation highly conserved across species, absent in gnomAD, and predicted damaging by multiple tools was classified as likely pathogenic, while benign profiles were categorized accordingly.

Participants found to harbor pathogenic or likely pathogenic variants received genetic counseling and were advised on cascade screening for family members and early CVD intervention strategies.

### Implementation to ensure validity and reliability

To ensure data validity and reliability, a series of rigorous procedures were implemented. First, the study protocol and data collection tools were pilot-tested to enhance clarity, and all clinicians and technicians underwent training on standardized clinical and anthropometric measurement methods, as well as phlebotomy and sample handling [46]. Anthropometric assessments were consistently performed by the same observer or pair of observers to reduce inter-observer variability, achieving a high intraclass correlation (>0.95) for BMI measurements. Standard operating procedures ensured specimen processing within two hours of collection, and biochemical assays were batch-processed under stringent internal quality control; blinded duplicates constituted 5% of samples, demonstrating reproducibility (CV for duplicate LDL-C measures was < 3%).

In genomic analysis, rigorous bioinformatics quality controls were applied. Sequencing achieved >100× average coverage with >99% of targeted bases covered ≥ 20×. Variants were filtered to minimize false positives by applying thresholds for read depth and alternate allele support. Critical variants (in *LDLR*,* APOB*, and *PCSK9*) were manually reviewed using the Integrative Genomics Viewer (IGV), with low-confidence variants confirmed via Sanger sequencing if necessary. To enhance data integrity, double data entry was employed for clinical and laboratory information, achieving an initial error rate below 0.5%, corrected through cross-verification [46].

Range and logic checks were incorporated into the database to flag anomalies. During data cleaning, blinding to group allocation was maintained to prevent bias. Additionally, laboratory personnel conducting genetic analyses and clinical geneticists classifying variants were blinded to the CVD status of participants. Regular team meetings monitored data quality and adherence to Good Clinical Practice standards. These comprehensive quality measures were essential for ensuring reproducibility and trustworthy comparisons between FD patients with and without CVD.

### Study dependent and independent variables

The primary outcome of this study was cardiovascular disease (CVD) status: Patients were divided into two groups: Group 1 (FD + CVD) and Group 2 (FD without CVD).

Key independent variables were clinical and biochemical measures known to influence CVD risk in FD patients, including age, sex, LDL-C, HDL-C, triglycerides, BMI, blood pressure, smoking status, and presence of xanthomas, selected based on prior associations with CVD risk in FH [37]. Additionally, family history of premature CVD was recorded. Lipid levels were considered both as continuous and categorical variables (e.g., LDL-C ≥ 190 mg/dL). When available, baseline untreated values were used.

In the sequenced subset, patients were classified as either “genotype-positive” (presence of pathogenic or likely pathogenic variants) or “genotype-negative.” An exploratory analysis considered variant pathogenicity tiers. Cardiovascular events were adjudicated by a blinded cardiologist, and genetic variant classifications were performed independently of clinical outcomes.

### Composite risk score construction and justification

To assess cardiovascular risk among FD patients carrying missense variants, a 10-variable composite risk score was developed. The composite risk score integrated the clinical, biochemical, genetic, and in silico bioinformatics data.

The selected predictors and their rationale are as follows:

1. *ACMG Variant Classification: *Pathogenic or likely pathogenic variants (scored as 1) indicate direct functional impairment [41, 47].
2. *Zygosity:* Homozygous variants (2) typically present with more severe phenotypes; heterozygous variants (1) carry intermediate risk [48].
3. *ΔΔG Stability Score: *A destabilizing ΔΔG ≤ − 1.5 kcal/mol (1) was included as it reflects the predicted loss of protein function; neutral variants scored 0 [42, 43].
4. *LDL-C Levels:* Levels ≥ 190 mg/dL (1) is well-established markers of elevated atherosclerotic risk [49].
5. *TG/HDL Ratio:* Used as a continuous variable; elevated ratios correlate with insulin resistance and CVD [50–52].
6. *BMI: *Categorized as ≥ 27 (1) vs. <27 (0), based on CVD risk thresholds in lipid disorder populations [53].
7. *Xanthelasma or Tendon Xanthomas:* Physical signs of lipid accumulation (scored as 1 if present) [54].
8. *PolyPhen-2 Prediction: *Scores ≥ 0.85 indicate probable damage (1); <0.85 scored as 0 [43, 55].
9. *SIFT Score: *<0.05 predicted to be deleterious (1); ≥0.05 considered tolerated (0) [56, 57].
10. *CADD Score:* Scores ≥ 20 (1) identify variants in the top 1% most deleterious [58].

These variables were selected based on robust literature evidence linking each to lipid metabolism and cardiovascular risk. Binary and scaled inputs were summed to yield a composite risk score ranging from 0 to ≥ 7.

To establish meaningful clinical thresholds, receiver operating characteristic (ROC) analysis was applied to determine optimal cutoffs. Based on empirical distribution and diagnostic performance metrics, risk categories were defined as: Low Risk (score 0–3), Moderate Risk (4–6), and High Risk (≥ 7).

A parallel model excluding bioinformatics variables was also tested to evaluate diagnostic utility in settings where genetic analysis may be limited.

### Statistical analysis

All analyses were conducted using IBM SPSS Statistics for Windows, version 26.0 (IBM Corp., Armonk, NY, USA). Descriptive statistics summarized participant characteristics. The Shapiro–Wilk test assessed normality; and variables were treated accordingly: normally distributed data were compared using independent-samples t-tests, while non-normally distributed data employed Mann–Whitney U tests. For categorical comparisons, z test between proportions and chi-square test were used; Fisher’s exact test was applied when cell counts were small. Genotype–phenotype correlations were evaluated using the same statistical tests applied to other study variables according to data type. ROC curve analysis was applied, achieving an area under the curve (AUC) of ~ 0.80. Significance was set at *p* < 0.05.

### Clinical characteristics

Among the 60 patients diagnosed with FD, 30 had CVD and 30 did not were compared. The two groups were similar in age and BMI, with no statistically significant differences observed (*p* = 0.638 and *p* = 0.252, respectively). However, the proportion of males was significantly higher in the non-CVD group (70.0%) compared to the CVD group (43.3%) (*p* = 0.037), suggesting a potential sex-based difference in CVD susceptibility. Triglyceride levels were significantly elevated in the CVD group (median: 356.5 mg/dL, IQR: 307.0–411.0) compared to the non-CVD group (median: 236.5 mg/dL, IQR: 166.8–262.5), with a highly significant p-value (< 0.001). In contrast, total cholesterol, HDL, and LDL levels showed no significant differences between groups, although LDL levels were numerically higher in the CVD group (460.0 mg/dL vs. 288.5 mg/dL, *p* = 0.290). These findings are summarized in Table 1.

**Table 1:** Demographic and clinical characteristics of familial dyslipidemia (FD) patients with and without cardiovascular disease (CVD)

**Characteristic**	**FD with CVD (n = 30)**	**FD without CVD (n = 30)**	**p-value**
Age (years), mean ± SD	33.2 ± 8.2	32.2 ± 9.5	0.638
Range	21–50	18–55	
Male, n (%)	13 (43.3 %)	21 (70.0 %)	0.037 *
BMI (kg/m²), mean ± SD	24.8 ± 2.1	24.2 ± 1.9	0.252
Range	22.1–29.4	20.3–28.9	
Triglycerides (mg/dL), median (IQR)	356.5 (307.0–411.0)	236.5 (166.8–262.5)	< 0.001 **
Range	210.0–680.0	110.0–531.0	
Total Cholesterol (mg/dL), median (IQR)	350.5 (301.3–392.0)	378.0 (290.0–597.5)	0.301
Range	250.0–816.0	205.0–912.0	
HDL (mg/dL), median (IQR)	65.0 (40.8–72.8)	65.5 (38.7–83.5)	0.700
Range	21.0–267.0	30.0–264.0	
LDL (mg/dL), median (IQR)	460.0 (203.8–691.0)	288.5 (206.3–564.5)	0.290
Range	125.0–786.0	56.0–806.0	

*FD* Familial dyslipidemia,* CVD* Cardiovascular disease, *BMI* Body mass index,* HDL* High-density lipoprotein, *LDL* Low-density lipoprotein, *IQR* Interquartile range

Statistical tests used: Independent t-test (means), Mann–Whitney U test (medians), and Chi-square test (proportions)

*Significant at p < 0.05

**Highly significant at p < 0.01

### Molecular genetic variants

Table 2 shows the summary of genetic variants detected in FD patients with and without cardiovascular disease (CVD). Genetic analysis revealed distinct patterns between the two groups. The *LDLR* gene showed significant differences: homozygous pathogenic *LDLR* variants were found in 26.7% of FD patients without CVD but in none of the CVD patients (p = 0.002), while heterozygous pathogenic LDLR variants were more frequent in the CVD group (30.0% vs. 3.3%, p = 0.006). Similarly, heterozygous likely pathogenic LDLR variants were observed in 43.3% of FD patients with CVD, compared to 20.0% in the non-CVD group (p = 0.05). Other *LDLR *mutations, such as c.1999T>C/G, c.2389G>A, c.2416dup, appeared at similar frequencies in both groups (16.7% vs. 13.3%, p = 0.719). A *LDLR*variant of uncertain significance (VUS) (c.2552A>T) was seen only in the non-CVD group (6.7% vs. 0%, p = 0.15). Rare LDLR large alterations showed no significant group differences.

**Table 2:** Summary of genetic variants detected in FD patients with and without CVD

Gene	Variant(s) Identified	Zygosity	ACMG Classification	FD without CVD (*n* = 30)	FD with CVD (*n* = 30)	*P* value
***LDLR*** (NM_000527.2)	c.501 C > A, c.1463T > C, c.1846-1G > A	Homozygous	Pathogenic	8 (26.7%)	0 (0.0%)	0.002**
	c.502G > A, c.1255T > G, c.1721G > A, c.1731G > T, c.1757 C > G	Heterozygous	Pathogenic	1 (3.3%)	9 (30.0%)	0.006**
	c.588del, c.907 C > T, c.1217G > A, c.1301 C > A, c.1659 C > G, c.1727 A > C	Heterozygous	Likely Pathogenic	6 (20.0%)	13 (43.3%)	0.05
	c.1999T > C, c.1999T > G, c.2389G > A, c.2416dup	Homozygous	Pathogenic/Likely Pathogenic	5 (16.7%)	4 (13.3%)	7.19
	c.2552 A > T	Heterozygous	VUS	2 (6.7%)	0 (0.0%)	0.15
	chr19:11,221,376–11,221,383	Homozygous	Likely Pathogenic	2 (6.7%)	0 (0.0%)	0.15
	chr19:11210885–11240361	Heterozygous	Pathogenic	0 (0.0%)	1 (3.3%)	0.31
***APOB*** (NM-000384.2)	c.3740 A > G, c.12137G > A	Heterozygous	VUS	2 (6.7%)	1 (3.3%)	0.56
	c.10142T > A	Heterozygous	Likely Pathogenic	4 (13.3%)	1 (3.3%)	0.16
***PCSK9*** (NM_174936.4)	c.1487G > A	Heterozygous	VUS	0 (0.0%)	1 (3.3%)	0.31

*FD *Familial Dyslipidemia, *CVD* Cardiovascular Disease, *LDLR* Low-Density Lipoprotein Receptor, *APOB* Apolipoprotein B,* PCSK9* Proprotein Convertase Subtilisin/Kexin Type 9, *ACMG* American College of Medical Genetics and Genomics, *VUS* Variant of Uncertain Significance

Statistical test used: z test between FD without CVD & FD with CVD proportions

**Highly significant at *p* < 0.01

In the *APOB* gene, heterozygous VUS variants were found in similar frequencies in both groups (6.7% in non-CVD vs. 3.3% in CVD; p = 0.56), while the likely pathogenic APOB variant (c.10142T>A) was more common in FD patients without CVD (13.3% vs. 3.3%, p = 0.16). A *PCSK9 *variant (c.1487G>A) of uncertain significance was found in only one FD patient with CVD (3.3%) and none without (p = 0.31). Overall, *LDLR* pathogenic variants were the most prevalent and strongly associated with CVD, while* APOB *and *PCSK9 *variants showed no significant difference between groups. Supplementary Table S1 provides detailed information on all variants identified in the study, including gene, transcript ID (RefSeq), cDNA and protein changes (HGVS), variant type, dbSNP ID (“rs” number), and clinical significance for each case examined.

### Genotype–phenotype correlations

Genotype–phenotype correlation in table 3 shows distinct clinical trends across the three major genes implicated in FD: *LDLR, APOB, *and* PCSK9*. In this cohort of 60 FD patients, *LDLR* variants were more frequent in the CVD subgroup compared with the non-CVD subgroup (27 (90.0%) vs 24 (80.0%). Whereas*APOB* variants were less common (6.7% vs 20%) and *PCSK9* variants were rare (3.3% vs 0%). There was no statistically significant difference in these genotype frequencies between the non-CVD and CVD groups (e.g. *LDLR* 90% vs 80%, p = 0.28; *APOB* 6.7% vs 20%, p = 0.13).

**Table 3:** Genotype–Phenotype correlation in FD patients with vs. without CVD

Gene	Representative Variants	TG median (IQR*) (mg/dL)	LDL median (IQR*) (mg/dL)	BMI mean ± SD (kg/m²)	Xanthelasma (*n* = 33) *N* (%)	FD without CVD (*n* = 30)	FD with CVD (*n* = 30)	*P* value
***LDLR*** (NM_000527.2)	c.501 C > A, c.1463T > C, c.1846-1G > A c.502G > A, c.1255T > G, c.1721G > A, c.1731G > T, c.1757 C > G c.588del, c.907 C > T, c.1217G > A, c.1301 C > A, c.1659 C > G, c.1727 A > C c.1999T > C, c.1999T > G, c.2389G > A, c.2416dup c.2552 A > T chr19:11,221,376–11,221,383 chr19:11210885–11240361	289.0 (220.0–386.0.0.0)	388.0 (205.0–640.0.0.0)	24.5 ± 2.1	29 (87.9%)	24 (80.0%)	27 (90.0%)	0.28
***APOB*** (NM-000384.2)	c.3740 A > G, c.12137G > A c.10142T > A	275.5 (252.5–446.0)	260.0 (165.4–287.8.4.8)	24.1 ± 1.9	0 (0.0%)	2 (6.7%)	6 (20.0%)	0.13
***PCSK9*** (NM_174936.4)	c.1487G > A	210.0 (210.0-210.0.0.0.0.0.0.0)	230.0 (230.0-230.0.0.0.0.0.0.0)	24.5 ± 0.0	4 (12.1%)	1 (3.3%)	0 (0.0%)	0.31

Percentages represent the proportion of variant carriers with xanthelasma or cardiovascular disease (CVD). Values are approximate summaries derived from variant-level data. Statistical test used: z test between FD without CVD & FD with CVD proportions

*TG* Triglycerides, *LDL* Low-Density Lipoprotein, *BMI* Body Mass Index

*LDLR* variant carriers exhibited the highest median levels of both triglycerides (TG: 360.0 mg/dL) and low-density lipoprotein cholesterol (LDL: 555.0 mg/dL), with a mean BMI of 24.5 ± 1.9 kg/m². Among *LDLR* variant carriers, the prevalence of xanthelasma and cardiovascular disease (CVD) was also elevated (9.0% and 9.2%, respectively), indicating a strong phenotype–genotype association. In contrast, carriers of *APOB* variants showed more moderate lipid abnormalities, with a median TG of 280.0 mg/dL and LDL of 230.0 mg/dL. Their mean BMI was slightly lower (23.6 ± 1.8 kg/m²), and the frequencies of xanthelasma (4.5%) and CVD (1.7%) were substantially reduced compared to LDLR. Only one variant in PCSK9 was observed, with corresponding lipid values (TG: 210.0 mg/dL, LDL: 230.0 mg/dL) and BMI (24.5 ± 0.0 kg/m²) falling within a similar range to APOB, while xanthelasma was absent and CVD was present in 3.3% of cases. Overall, no p < 0.05 differences were observed between the CVD vs non-CVD groups in these genotype-specific phenotypic measures from Table 3.

### Functional predictions of missense variants

Table 4 summarize the predicted functional impact of missense variants in three key genes; *LDLR*, *APOB*, and *PCSK9* using combined bioinformatics scores from SIFT, PolyPhen-2, and CADD. The majority of *LDLR* variants (n = 8) were predicted to be deleterious across all three scoring systems (SIFT < 0.05, PolyPhen-2 >0.85, and CADD ≥ 20), and these were disproportionately present in FD patients with CVD (56.7%) compared to those without (10 %). Additionally, four *LDLR* variants had uncertain or mixed predictions, showing a more balanced distribution between the two groups (10 % vs. 13.3). In contrast, *APOB* variants were mostly classified as benign or of uncertain significance, and were evenly distributed between FD patients with and without CVD. A single *PCSK9* variant was identified and was classified as benign based on all scoring tools; it appeared only in one patient with CVD. Supplementary Table S2 lists all missense variants analyzed, including the eight deleterious LDLR variants and other benign or uncertain variants with detailed functional annotation and predicted effects derived from multiple in silico tools (SIFT, PolyPhen-2, CADD, ΔΔG, and RI scores).

**Table 4:** Summary of predicted functional impact of missense variants

Gene	Pathogenicity (by SIFT/PolyPhen-2/CADD)	Number of Variants	FD with CVD (*n* = 30)	FD without CVD (*n* = 30)	*P* value
*LDLR*	Deleterious (SIFT < 0.05, PolyPhen > 0.85, CADD ≥ 20)	8	17 (56.7%)	3 (10.0%)	< 0.001**
*LDLR*	Uncertain (mixed or borderline predictions)	4	3 (10.0%)	4 (13.3%)	0.69
*APOB*	Mostly benign or uncertain	2	1 (3.3%)	2 (6.7%)	0.56
*PCSK9*	Benign (SIFT > 0.05, PolyPhen < 0.15, CADD < 20)	1	1 (3.3%)	0 (0.0%)	0.31

*SIFT* Sort Intolerant From Tolerant, *PolyPhen-2* Polymorphism Phenotyping v2, *CADD* Combined Annotation Dependent Depletion. Deleterious variants meet all three thresholds: SIFT < 0.05, PolyPhen-2 > 0.85, and CADD ≥ 20. Statistical test used: z test between FD without CVD & FD with CVD proportions

**Highly significant at *p* < 0.01

### Protein stability analysis

Table 5 and supplementary Table S2 present an overview of predicted changes in protein stability due to missense variants in *LDLR*,*APOB*, and *PCSK9*, based on ΔΔG values generated by the I-Mutant tool. Most of the *LDLR* variants showed negative ΔΔG values, indicating decreased protein stability. Specifically, a cluster of *LDLR* variants (including c.907C>T, c.1217G>A, c.1727A>C, and c.1463T>C) had ΔΔG values ≤ −1.0, classifying them as destabilizing. These were predominantly found in patients with CVD (n = 17) versus a smaller number in those without CVD (n = 5). Other *LDLR* variants, such as c.502C>A and c.1301C>A, showed milder decreases in stability (ΔΔG around −0.1) and were more evenly distributed (FD with CVD = 8; without CVD = 3). Among the *APOB* variants, both c.3740A>G and c.12137G>A were predicted to be destabilizing but were found in only a few patients, mainly without CVD. The single *PCSK9* variant (c.1487G>A) also showed a destabilizing effect (ΔΔG = −1.84), and was observed in one patient with CVD.

**Table 5:** Summary of predicted protein stability changes (ΔΔG) using I-Mutant

Gene	Stability Effect	Representative Variants	Mean ΔΔG (kcal/mol)	FD with CVD (*n* = 30)	FD without CVD (*n* = 30)	*P* value
*LDLR*	Decrease/Destabilizing	c.907 C > T, c.1217G > A, c.1727 A > C, c.1463T > C	−1.8	17 (56.7%)	5 (16.7%)	0.001**
*LDLR*	Mild Decrease	c.502 C > A, c.1301 C > A, c.1731G > T	−0.1	8 (26.7%)	3 (10.0%)	0.09
*APOB*	Destabilizing	c.3740 A > G, c.12137G > A	−1.0	1 (3.3%)	2 (6.7%)	0.56
*PCSK9*	Destabilizing	c.1487G > A	−1.84	1 (3.3%)	0 (0.0%)	0.31

ΔΔG: Gibbs free energy change. A negative ΔΔG indicates decreased protein stability. Variants with ΔΔG ≤ − 1.0 were considered destabilizing; those with ΔΔG between − 1.0 and 0 were categorized as mild decrease. Predictions are based on I-Mutant 3.0 outputs. Statistical test used: z test between FD without CVD & FD with CVD proportions,

**Highly significant at *p* < 0.01

### Composite risk score performance

The composite risk panel stratified patients into moderate- and high-risk categories with respect to CVD. Most CVD patients (88.0%) were in the high-risk category, while more than half of the non-CVD patients (54.5%) were in the moderate-risk group (Table 6). No patients were categorized as low risk. The association between risk score category and CVD status was statistically significant (p = 0.007).

**Table 6:** Composite risk score categories and clinical implications among FD patients with missense variant

Composite Risk Score Category§	Total (*n* = 36)	FD with CVD (*n* = 25)	FD without CVD (*n* = 11)	*P* value	Clinical Implication
Low Risk (Score: 0–3) *P value by* z test	0 (0.0%)	0 (0.0%)	0 (0.0%)	0.012*	No current concern; routine monitoring sufficient
		**-------**			
Moderate Risk (Score: 4–6) *P value by* z test	9 (25.0%)	3 (12.0%)	6 (54.5%)		Consider routine follow-up and preventive interventions
		0.007**			
High Risk (Score: ≥7) *P value by* z test	27 (75.0%)	22 (88.0%)	5 (45.5%)		Initiate early intervention and intensive management
		0.007**			

§The composite risk score was computed based on a weighted algorithm incorporating clinical (e.g., BMI, triglyceride level, LDL, Cholesterol), biochemical (e.g., LDL-C, HDL-C), and genetic parameters (presence and pathogenicity of variants in LDLR, APOB, and PCSK9). Scores ranged from 0 to ≥ 7 and were categorized into three strata: low (0–3), moderate (4–6), and high (≥ 7). Statistical tests used: Fisher’s exact test between groups, z test between FD without CVD & FD with CVD proportions,

*Significant at *p* < 0.05, **Highly significant at *p* < 0.01

In FD patients carrying *missense* variants, a composite risk score panel was designed to differentiate those with cardiovascular disease (CVD) from those without (Table 7). The composite risk score panel was based on clinical, genetic and bioinformatics variables including ACMG Classification, Zygosity, ΔΔG (Stability Prediction), PolyPhen Prediction, SIFT Prediction, CADD Score, LDL Cholesterol, TG/HDL Ratio, BMI, and Xanthelasma/Tendon Xanthomas. The panel demonstrated moderate diagnostic performance in predicting CVD among FD patients with missense variants. The panel demonstrated high sensitivity (89.5%, 95% CI: 66.9%–98.7%), indicating its strong ability to identify patients with CVD correctly. However, the specificity was moderate at 52.9% (95% CI: 27.8%–77.0%), suggesting a moderate level of false positives among those without CVD. The positive predictive value (PPV) was 68.0 % (95% CI: 46.5%–85.1%), meaning two-thirds of patients predicted as high-risk were actually had CVD. The negative predictive value (NPV) was relatively strong at 81.8% (95% CI: 48.2%–97.7%), indicating that most patients classified as low risk were correctly predicted to be free of CVD. Overall diagnostic accuracy stood at 72.2% (95% CI: 54.8%–85.8%), suggesting that the model classified around 72% of the cases correctly. The p-value was statistically significant (p = 0.022), suggesting that the performance of the composite risk score was unlikely to be due to chance.

**Table 7:** Diagnostic performance of the composite risk score among Familial dyslipidemia patients having missense variants for predicting CVD

Metric§	Value	Interpretation
Sensitivity	89.5% (95%CI: 66.9%−98.7%)	High sensitivity indicates excellent ability to identify CVD-positive FD patients.
Specificity	52.9% (95%CI: 27.8%−77.0%)	Moderate specificity suggests some false positives in CVD risk classification.
Positive Predictive Value (PPV)	68.0% (95%CI: 46.5%−85.1%)	Two-thirds of those identified as high risk indeed had CVD.
Negative Predictive Value (NPV)	81.8% (95%CI: 48.2%−97.7%)	Strong ability to correctly rule out CVD in those categorized as low/moderate risk.
Accuracy	72.2% (95%CI: 54.8%−85.8%)	The model classifies about 7 in 10 cases correctly overall.
AUC (95% CI)	0.742 (95%CI: 0.55–0.93)	Indicates moderate-to-good discriminative performance.
P-value	0.022*	Statistically significant; performance unlikely due to chance (*p* < 0.05).

§Diagnostic metrics were calculated for a subset of 36 patients for whom complete composite risk scores and CVD status were available (Bioinformatics tools were applied only for missense variants). Factors in the model: ACMG Classification, Zygosity, ΔΔG, LDL Cholesterol, TG/HDL Ratio, BMI, Xanthelasma/Tendon Xanthomas, PolyPhen, SIFT and CADD Score

Sensitivity and specificity reflect the tool’s classification accuracy

**Fig. 1:**
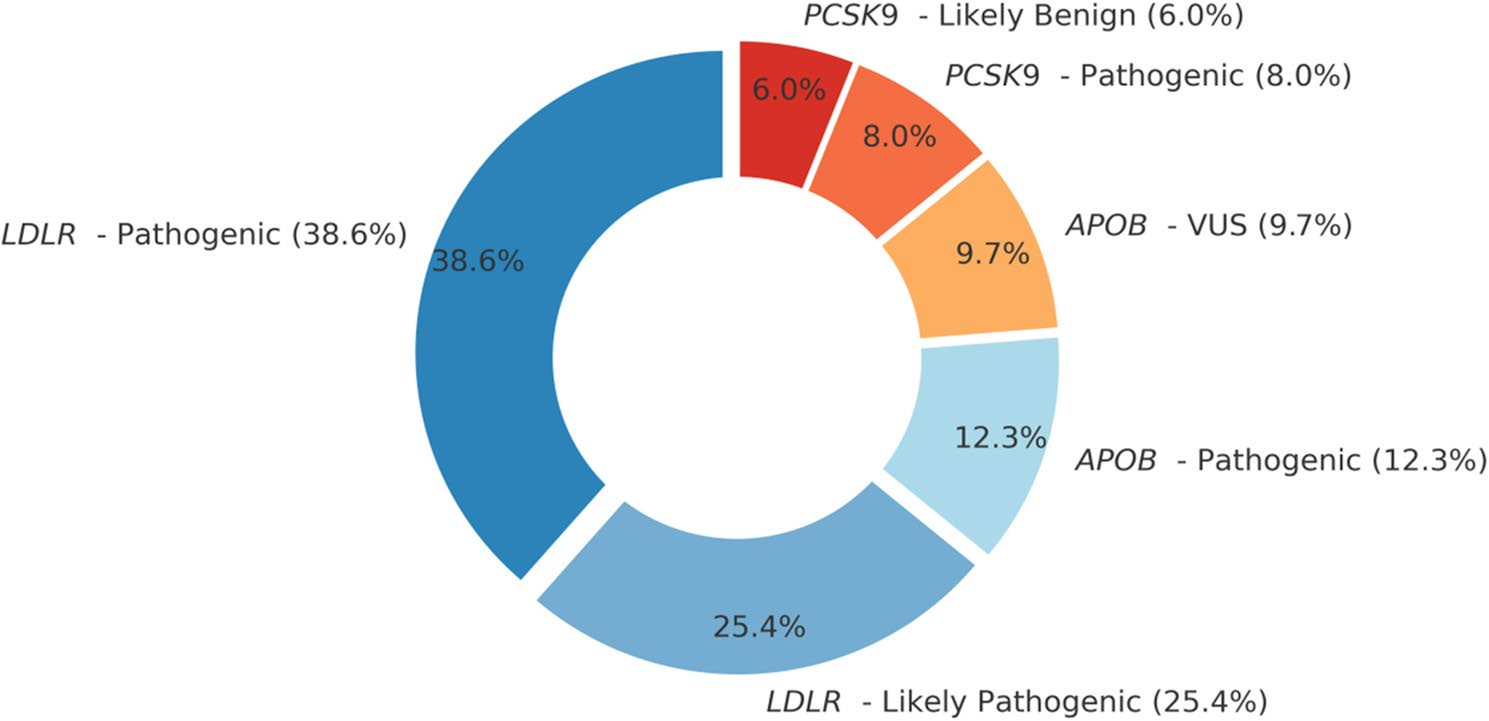
Distribution of identified variants by gene and pathogenicity class in the familial dyslipidemia cohort

*AUC* Area Under the Curve of the ROC plot shown in Figure 1

*Significant at <0.05

**Fig. 2:**
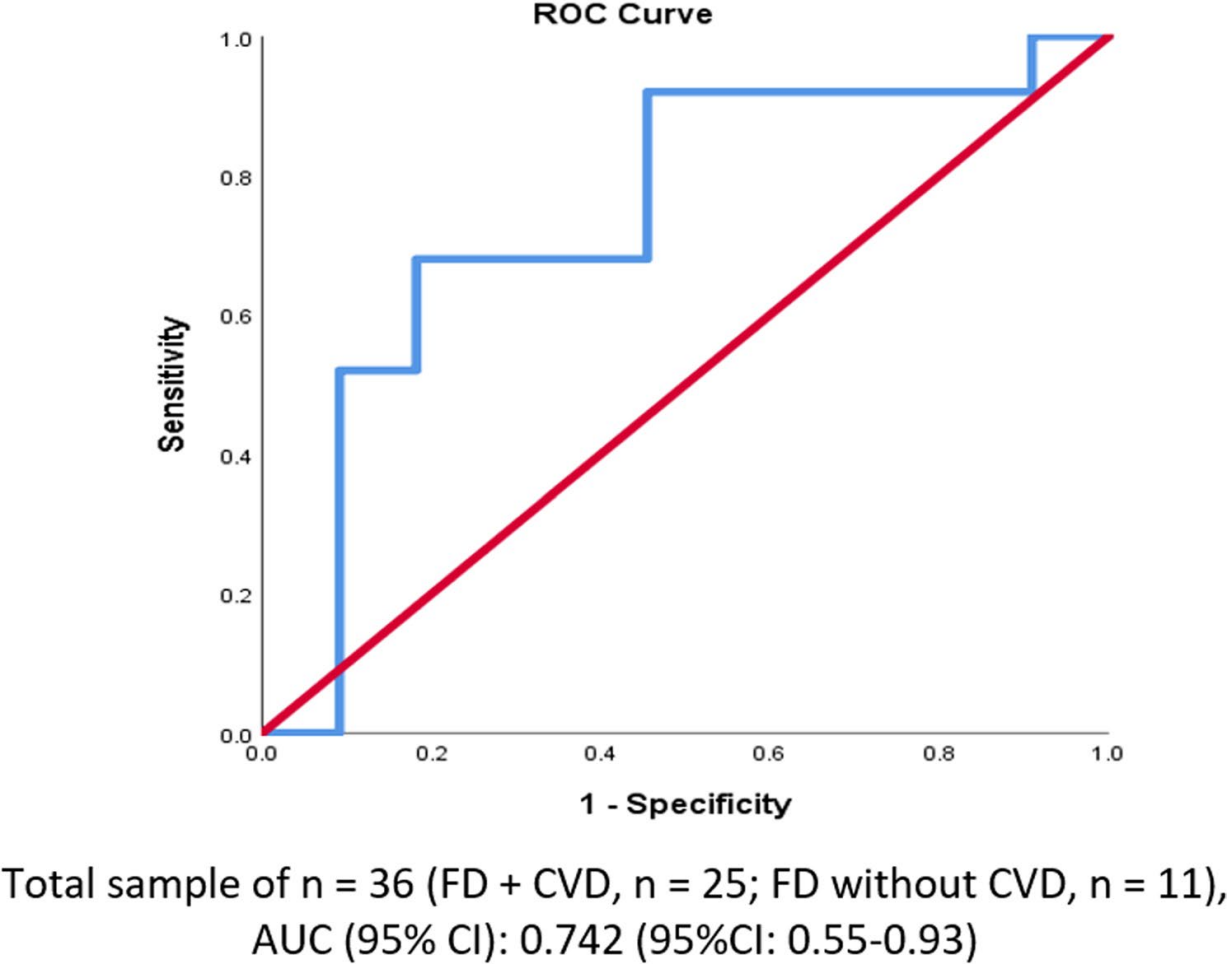
Receiver-Operating Characteristic (ROC) curve of the Composite Risk Panel model 1 for FD Patients with and without CVD

Figure 2 illustrates the ROC curve corresponding to the composite risk score applied to FD patients with missense variants for predicting CVD. The area under the ROC curve (AUC) was calculated at 0.742 (95% CI: 0.55–0.93), demonstrating moderate-to-good discriminative performance. The curve shape suggests a reasonable trade-off between sensitivity and specificity, with the model achieving high sensitivity (89.5%)and moderate specificity (52.9%), as noted in Table 7. The curve deviates clearly from the diagonal reference line, indicating better-than-random classification performance.

**Fig. 3:**
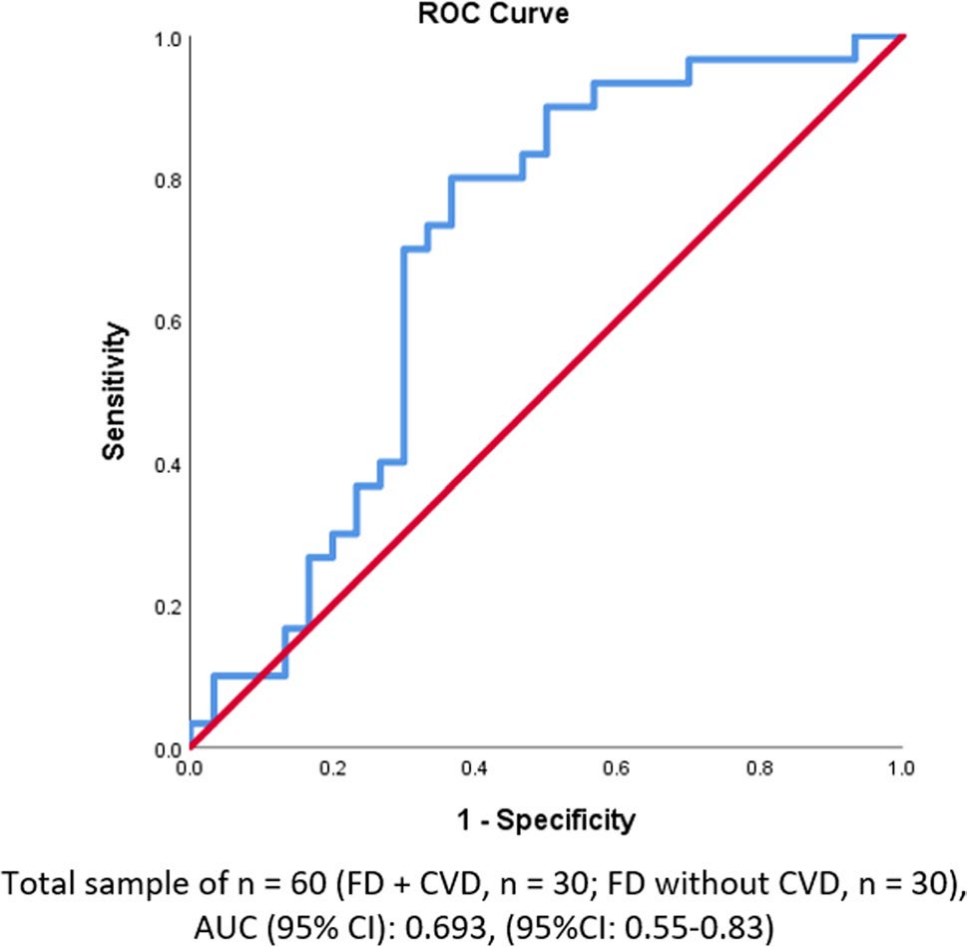
Receiver-Operating Characteristic (ROC) curve of the Composite Risk Panel model 2 for FD Patients with and without CVD

Table 8 shows the second diagnostic performance metrics of the composite risk score in predicting VD among FD patients. The composite risk score, based on clinical and genetic variables excluding Bioinformatics tools, while including ACMG Classification, Zygosity, LDL Cholesterol, TG/HDL Ratio, BMI, and Xanthelasma/Tendon Xanthomas, demonstrated moderate diagnostic performance in predicting CVD among FD patients. The sensitivity was 68.6%, indicating that it identified about 69% of true CVD cases, but missed a significant portion. The specificity was 76.0%, meaning the score correctly identified 76% of patients without CVD, but still flagged some healthy individuals as high-risk. The PPV was 80.0%, showing that most patients flagged as high-risk indeed had CVD. The NPV was 63.3%, indicating a notable chance that patients classified as low-risk could still have CVD. The overall accuracy was 71.7%, meaning the score correctly classified about 72% of patients. The area under the ROC curve (AUC) was 0.693, reflecting fair ability to distinguish between CVD and non-CVD patients. The results were statistically significant with a p-value of 0.010 (Figure 3).

**Table 8:** Diagnostic performance of the composite risk score in predicting CVD among FD patients

Metric§	Value	Interpretation
Sensitivity	68.6% (95%CI: 50.7%−83.1%)	The tool is **not highly sensitive** when trying to ensure all at-risk patients are caught early.
Specificity	76.0% (95%CI: 54.9%−90.6%)	Moderate specificity, has a **relative strength** in that it limits unnecessary worry or interventions for those truly disease-free,
Positive Predictive Value (PPV)	80.0% (95%CI: 61.4%−92.3%)	**High PPV**, the tool is effective at confirming risk, patients identified as high-risk by the score very often truly need cardiovascular attention or intervention.
Negative Predictive Value (NPV)	63.3% (95%CI: 43.9%−80.1%)	**Low NPV** is a significant limitation of the tool: a negative (low-risk) result is not very reassuring.
Accuracy	71.7% (95%CI: 58.6%−82.5%)	**Moderate overall accuracy**, Ca not rely solely on the tool
AUC (95% CI)	0.693 (95%CI: 0.55–0.83)	**Fair discriminative** ability of the risk score in distinguishing between FD patients with and without CVD.
P-value	0.010*	The risk score does have a real, measurable association with actual CVD outcomes in FD patients

§Diagnostic metrics were calculated for all patients with factors in the model: ACMG Classification, Zygosity, LDL Cholesterol, TG/HDL Ratio, BMI, Xanthelasma/Tendon Xanthomas

Sensitivity and specificity reflect the tool’s classification accuracy

AUC = Area Under the Curve of the ROC plot shown in Fig. 2

*Significant at < 0.05

## Discussion

This study provides novel insights into the genetic and clinical determinants of CVD risk among adolescents and young adults with FD in a high-consanguinity, resource-limited setting. By integrating next-generation sequencing, in silico functional annotation, and comprehensive clinical profiling, we present a multidimensional approach to risk stratification that extends beyond traditional lipid parameters. The development of a composite risk score tailored to variant type and phenotypic features represents a promising step toward precision cardiovascular prevention in genetically predisposed populations.

### Triglyceride significance and therapeutic implications

The findings of this study reveals that FD patients with CVD had significantly higher triglyceride levels compared to those without CVD (*p* < 0.001). Despite observed numerical trends, LDL-C and HDL-C did not significantly differ between patients with and without CVD. The association of elevated triglycerides with CVD supports growing evidence that hypertriglyceridemia contributes independently to atherogenesis in genetic lipid disorders [58–62]. These results emphasize the need to prioritize triglyceride-rich lipoproteins in cardiovascular risk management for FD patients. Elevated triglycerides contribute to endothelial dysfunction, pro-inflammatory states, and small dense LDL formation, all of which are atherogenic mechanisms [58, 59]. Triglyceride control may represent a critical therapeutic target for cardiovascular risk reduction in FD patients, especially those with residual risk despite statin therapy [63]. From a therapeutic perspective, these findings highlight the potential role of TG-lowering interventions in genetically predisposed subgroups. Fibrate therapy, via PPAR-α activation, enhances lipoprotein lipase activity and reduces hepatic VLDL synthesis, thereby mitigating TG-driven residual risk; particularly relevant to carriers of *APOB and PCSK9 *variants*. *Similarly, omega-3 polyunsaturated fatty acids (PUFAs), including EPA and DHA, have shown cardioprotective and anti-inflammatory properties that complement statin therapy and may modulate lipid–gene interactions through transcriptional and epigenetic pathways governing triglyceride metabolism[63–66] Integrating genotype-guided therapy with biochemical profiling could therefore represent a future direction in precision lipid management for FD.

### Sex-based differences and demographic insights

Interestingly, although males are typically at higher risk of CVD, the higher prevalence of females in the CVD group is notable and may reflect sex-specific susceptibility or disparities in healthcare access, diagnosis, or treatment; a phenomenon observed in other dyslipidemia cohorts [67]. This finding indicates a need for heightened CVD surveillance among female FD patients who may not present with traditional risk profiles. The predominance of females within the CVD subgroup in this adolescent and young-adult cohort may reflect the interplay of early hormonal and behavioral factors rather than menopause-related changes. Although estrogen is generally cardioprotective, metabolic stressors such as obesity, insulin resistance, and dyslipidemia can attenuate its favorable lipid and vascular effects [68–70]. Lifestyle factors, including lower physical-activity levels and suboptimal dietary habits, may further amplify triglyceride and HDL-C abnormalities among young females [71]. Additionally, sociocultural factors and lower preventive screening rates in young women could contribute to delayed detection and management of dyslipidemia [72, 73]. These findings highlight the importance of early, sex-sensitive interventions promoting healthy behaviors and equitable access to lipid screening in both sexes.

### Genetic variants in familial dyslipidemia and cardiovascular disease risk

Our study compared the distribution of genetic variants in the *LDLR*, *APOB*, and *PCSK9* genes among FD patients with and without CVD. The data reveal distinct genotype-phenotype correlations with important clinical implications.

The absence of homozygous pathogenic *LDLR* variants among CVD patients, despite a 26.7% presence in the non-CVD group, is both statistically significant and clinically noteworthy (*p* = 0.002). This finding may reflect early diagnosis and more aggressive lipid-lowering treatment in homozygotes, potentially delaying or preventing CVD onset in these individuals. Moreover, certain *LDLR* homozygous mutations may exhibit functional heterogeneity, with variable impacts on LDL receptor activity and atherogenicity [74, 75].

Interestingly, homozygous *LDLR* variants were observed more frequently among non-CVD participants, an apparently counterintuitive finding. This pattern may reflect age-related latency of cardiovascular manifestations, as most homozygous carriers in this cohort (15–25 years) were still below the typical age at which atherosclerotic complications become clinically evident [2]. Earlier diagnosis through family screening and more aggressive lipid-lowering therapy—often involving high-intensity statins and ezetimibe—could have mitigated progression to overt CVD [76, 77]. Moreover, mutation-specific functional differences may play a role: receptor-defective variants retaining partial *LDLR* activity produce a milder biochemical phenotype than receptor-negative mutations [78, 79]. Collectively, these factors may explain the higher representation of homozygous variants in the non-CVD subgroup and underscore the heterogeneity of clinical expression in familial dyslipidemia.

Conversely, the significantly higher frequency of heterozygous pathogenic and likely pathogenic *LDLR* variants in the CVD group (*p* = 0.006 and *p* = 0.05, respectively) suggests that even a single defective allele confers substantial CVD risk if left untreated or insufficiently controlled. This aligns with data from the CASCADE FH Registry and other cohort studies, which have shown that heterozygous familial hypercholesterolemia (HeFH) patients remain at increased risk of premature coronary events despite modest lipid elevations [80–82].

Variants in *APOB* and *PCSK9* were less prevalent and not statistically different between groups, consistent with previous literature indicating their lower penetrance compared to *LDLR* mutations [83–85]. However, the presence of likely pathogenic *APOB* variants in non-CVD patients supports a milder clinical phenotype often observed in APOB-associated hypercholesterolemia, marked by lower LDL-C burden and fewer xanthomas [86]. Nevertheless, CVD events in some *APOB* mutation carrier emphasise that moderate, lifelong LDL elevation can still promote atherosclerosis if untreated [51].

The identification of VUS in both groups reflects ongoing challenges in variant interpretation. These findings highlight the need for cautious genetic counseling and the integration of functional studies, family segregation, and bioinformatics prediction tools into variant classification frameworks [41].

In clinical practice, these data reinforce the utility of genetic testing not only for diagnostic confirmation but also for risk stratification. The presence of heterozygous *LDLR* mutations in CVD patients underlines the importance of early detection and intensive LDL-lowering therapy, even in individuals without overt xanthomas. Integrating genotype data with clinical phenotypes enables precision management of FD and targeted family screening strategies, in line with current international guidelines [62]. To contextualize these findings regionally, comparative data from other Middle Eastern and Mediterranean familial dyslipidemia and FH registries were reviewed. Comparative insights from neighboring Middle Eastern and North African cohorts reveal both shared and population-specific mutation patterns. Registries from Saudi Arabia, Tunisia, and Lebanon have reported recurrent *LDLR* and *APOB* variants such as *LDLRp.G592E, p.C681X,* and *APOB p.R3500Q*, which also appear in Mediterranean and North African datasets, suggesting common founder lineages [10, 87–89]. Conversely, locally restricted *LDLR *splice-site and missense variants; often within the ligand-binding and EGF-precursor domains reflect the effects of consanguinity and regional genetic drift [75, 90]. These regional parallels reinforce the need for population-tailored variant databases and emphasize how our Egyptian cohort contributes to refining genomic reference data for the broader MENA region.

### Genotype–phenotype correlations

The study findings highlight important genotype–phenotype relationships and genetic insights in FD. Notably, *LDLR* mutations predominated in this cohort, consistent with the fact that *LDLR* defects account for the majority (85–90%) of familial hypercholesterolemia cases [91]. *LDLR* mutations lead to more severe hyperlipidemia and clinical signs compared to *APOB* or *PCSK9* mutations. The *LDLR* variant carriers had dramatically elevated LDL-C (median near 388 mg/dL) and frequently developed xanthelasma, reflecting longstanding extreme cholesterol levels. In contrast*, APOB* mutation carriers showed lower median LDL-C and **no** xanthelasma, consistent with the known milder effect of *APOB* variants on LDL metabolism [92]. Importantly, however, the lack of a statistically significant difference in mutation frequency between FD patients with and without CVD (despite *LDLR* carriers tending to have higher absolute LDL levels) suggests that possessing an *LDLR* vs *APOB* mutation was not, in itself, determinative of CVD occurrence in this sample. This could be due to high baseline LDL-C in all patients (even *APOB *carriers had very elevated LDL levels) and effective management or other factors mitigating risk in some *LDLR* cases. There was a trend toward a higher proportion of *APOB* mutations in the CVD group (20% vs 6.7%), indicating that even the “milder” familial hypercholesterolemia from *APOB* variants can lead to premature CVD if not adequately treated or if other risk factors are present. Clinically, the markedly higher LDL-C associated with *LDLR* mutations (50% higher median LDL than seen in *APOB* carriers) highlights the need for especially aggressive lipid-lowering therapy in *LDLR*-related FH to prevent atherosclerosis [93]. The strong association of xanthelasma with LDLR mutations highlights that such physical stigmata signal a high lifelong LDL burden and correlate with elevated cardiovascular risk [93]. By contrast, APOB mutation carriers lacked xanthelasma, implying a lower cumulative LDL exposure, yet those who did develop CVD demonstrate that even moderately severe lifelong hypercholesterolemia can cause arterial disease over time [74]. Finally, the similar normal-range BMI (4 kg/m²) in both CVD and non-CVD groups suggests that obesity was not a confounding driver of CVD in this cohort; the risk was driven by genetic dyslipidemia itself.

The presence of multiple VUS, particularly in *APOB*, highlights the challenges of interpreting rare or novel mutations, emphasizing the importance of integrating computational tools, population allele frequencies, and functional studies [41, 94]. The identification of a *PCSK9* variant in a CVD patient, although isolated, aligns with its known regulatory function in LDL receptor degradation and warrants further exploration [95, 96].

Genotype–phenotype correlations revealed that *LDLR* variants were associated with more severe biochemical profiles and a higher frequency of clinical manifestations, including xanthelasma and CVD, reinforcing its central pathogenic role in monogenic dyslipidemias [75]. By contrast, *APOB* variants were associated with milder lipid abnormalities and less consistent clinical outcomes, in line with their known variable penetrance [78]. Although limited in number, the *PCSK9* variant appeared in a patient with CVD but without xanthelasma, which is consistent with the gene’s role in receptor turnover rather than lipid transport [75]. These findings highlight the importance of integrating genetic data with clinical and biochemical profiles to identify high-risk subgroups and guide personalized interventions [62, 97].

### Predicted functional impact of missense variants

The current study demonstrates that deleterious missense variants in the *LDLR *gene; predicted by combined SIFT, PolyPhen-2, and CADD scores were significantly more prevalent among FD patients with CVD compared to those without CVD (56.7% vs. 10.0%, p < 0.001). These results emphasize the critical role of *LDLR* deleterious missense variants in the pathogenesis of cardiovascular disease among FD patients and highlight the functional relevance of bioinformatically predicted variant severity in risk stratification. Variants that met all three damaging thresholds (SIFT, PolyPhen-2, and CADD) likely contribute to impaired LDL receptor activity, resulting in greater LDL accumulation and accelerated atherosclerosis if left untreated [41, 75, 81].

By contrast, *APOB* and *PCSK9* variants demonstrated low predictive burden and did not significantly differ by CVD status. This aligns with previous reports indicating that *LDLR* mutations are the primary genetic drivers in FH, while *APOB* and *PCSK9* mutations often result in milder phenotypes or require additional modifiers to manifest disease [83, 86, 98].

Importantly, the study illustrates the utility of incorporating multi-algorithm bioinformatics scoring into clinical genetics. Combined pathogenicity thresholds; particularly a CADD score ≥20, SIFT < 0.05, and PolyPhen-2 >0.85 have been shown to reliably enrich for disease-causing variants in monogenic lipid disorders [99, 100].Our findings support the use of bioinformatics annotation as a practical and informative strategy to interpret missense variants when functional assays are not feasible. Similar strategies have been effectively applied in Egyptian genomic research to expand phenotype–genotype correlations [101]. Their use in variant interpretation enhances diagnostic precision, especially for missense changes where empirical data may be lacking.

These findings support integrating in silico tools with genotype-phenotype analyses to optimize CVD risk prediction and cascade screening strategies in FD families, in accordance with NICE and ACC recommendations for genetic testing and variant classification in FH [62, 102]. In future work, experimental validation will be pursued through quantitative LDL receptor (*LDLR*) activity assays and plasma apolipoprotein B and *PCSK9* measurements to confirm in silico predictions and refine genotype–phenotype correlations.

### Predicted protein stability changes (ΔΔG) and their association with cardiovascular risk

The current study demonstrated that destabilizing mutations in the *LDLR* gene (ΔΔG ≤ −1.0 kcal/mol) were significantly more common among FD patients with CVD compared to those without (56.7% vs. 16.7%, *p* = 0.001), whereas destabilizing variants in *APOB* and *PCSK9* were infrequent and showed no significant group differences. These results highlight the pathogenic potential of *LDLR* variants that significantly destabilize protein structure, particularly in FD patients with CVD. The higher prevalence of destabilizing *LDLR* mutations in this group supports the hypothesis that such variants contribute to impaired receptor function, reduced LDL clearance, and accelerated atherogenesis [79, 81]. This finding is consistent with prior work linking specific *LDLR* mutations to structural disruption and severe phenotypes in familial hypercholesterolemia (FH) [103]. In contrast, *APOB* and *PCSK9* destabilizing variants were infrequent, aligning with their typically lower penetrance and more variable impact on lipid profiles [104, 105]. Bioinformatic tools such as I-Mutant 3.0, when integrated with clinical phenotype, offer valuable insights for variant classification and cardiovascular risk stratification in FD populations [43]. The inclusion of ΔΔG in composite risk models may improve the identification of high-risk genotypes warranting intensive management.

### Risk stratification using composite score among FD Patients with missense variants

The study showed that the majority of FD patients with missense variants who developed CVD were classified as high risk (88.0%) based on the composite score, whereas over half of those without CVD (54.5%) fell into the moderate-risk category, indicating the score’s strong discriminative performance (p = 0.007). The current data validated the clinical utility of a composite risk scoring system that integrates genetic, clinical, and biochemical factors; including missense variant pathogenicity, LDL-C, BMI, and triglyceride/HDL ratio to stratify cardiovascular risk in FD patients. The absence of low-risk individuals and the concentration of high-risk scores among patients with CVD support previous findings that cumulative risk from multiple moderate predictors (e.g., mildly pathogenic variants, borderline lipid levels) can reach a clinically actionable threshold when integrated [62, 74]. Importantly, the finding that over half of the FD patients without CVD fell into the moderate-risk category suggests that this group could benefit from preventive strategies to mitigate future CVD risk, aligning with international guidelines for early risk-based intervention in genetically predisposed populations [102, 106].

### Composite risk score performance with emphasis on sensitivity, NPV, and specificity

The enhanced performance of the missense-only subgroup appears driven by the inclusion of genetic and bioinformatics variables; such as CADD score, ΔΔG, and PolyPhen prediction. These tools have been shown to improve the sensitivity and negative predictive value (NPV) of variant classification and risk models, making them effective at identifying individuals without disease and ruling out CVD when scores are low [100, 107].

Clinically, this finding has significant implications in FD. In missense variant carriers, the composite risk score achieved high sensitivity (89.5%) and NPV (81.8%), indicating its strength in correctly identifying true CVD cases and reliably excluding those without disease. High sensitivity minimizes the chance of missing at-risk individuals; crucial for early detection in genetically predisposed populations, while strong NPV supports confident deferral of invasive evaluation in low-risk patients [108, 109].

However, specificity (52.9%) and positive predictive value (PPV, 68.0%) were comparatively lower in the missense group, leading to more false-positive results. This suggests the score is better suited as a rule-out tool rather than for definitive diagnosis. As such, positive findings should prompt follow-up confirmatory testing rather than immediate therapeutic action [108, 109].

In contrast, within the broader FD cohort, the score showed higher specificity (76.0%) and PPV (80.0%), meaning it was more effective at confirming true CVD cases. However, sensitivity (68.6%) and NPV (63.3%) were reduced, indicating the risk of missing true cases. Therefore, when applied to the general FD population, the score should be supplemented with other diagnostic tools to avoid under-detection of at-risk individuals [108].

Thus, the composite risk score appears to be highly sensitive in missense-variant FD patients and useful for excluding CVD when risk is low, but its propensity for false positives and the uncertainty in the estimates (due to wide CIs) warrant caution. Clinically, this means the tool could be very helpful for screening (to catch as many CVD cases as possible among those with risky variants) and for reassuring low-risk patients, but a positive/high-risk result should be interpreted in context; possibly confirmed with further testing or risk assessment given the moderate PPV and specificity.

**Fig. 4:**
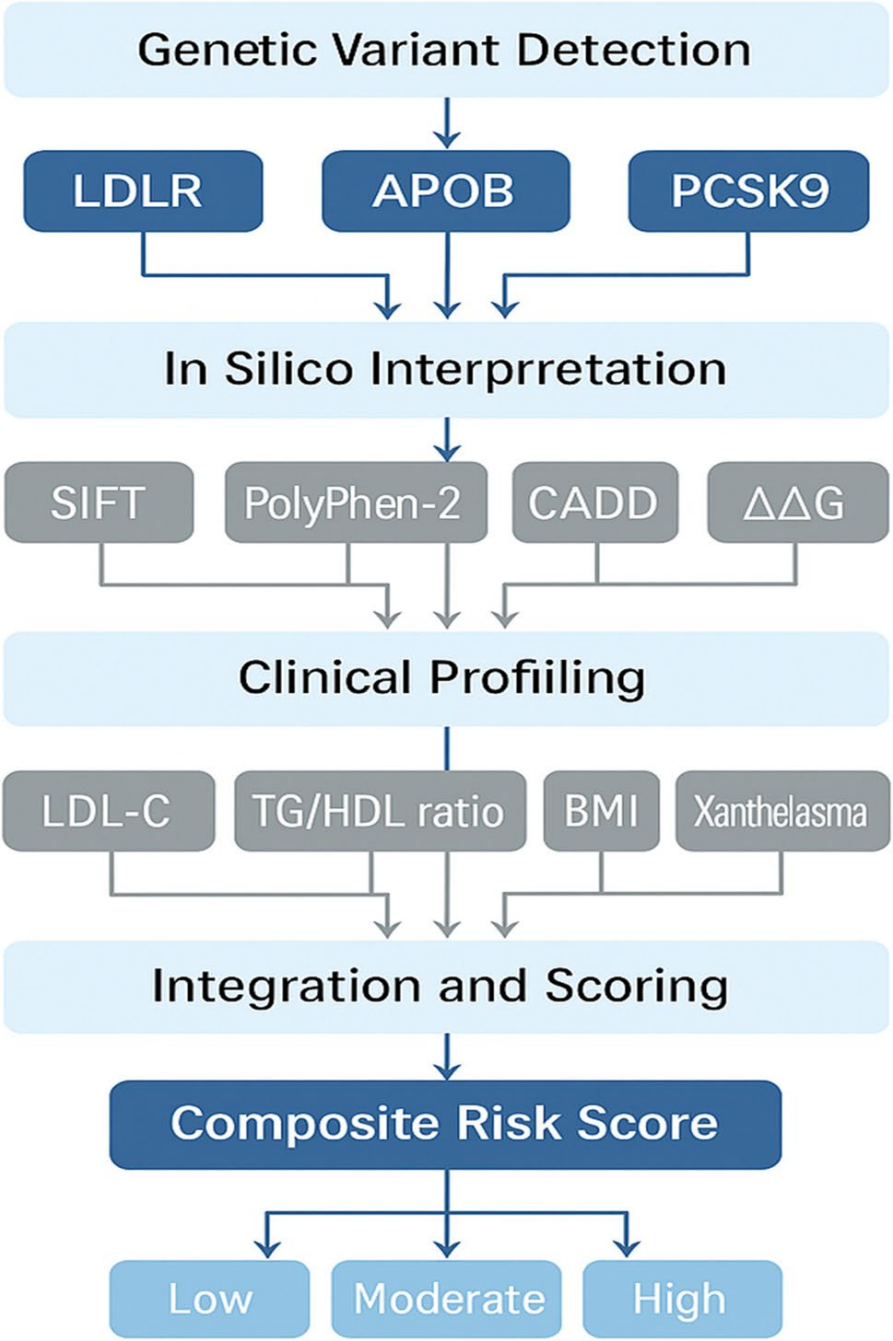
Genotype-phenotype relationship and composite risk score for CVD in FD

Overall, this supports a two-tiered, precision approach: using the composite score with bioinformatics-enhanced sensitivity to screen and exclude low-risk individuals in the missense subgroup, while leveraging improved specificity in broader FD populations to confirm CVD risk prior to intervention. To aid visual understanding, a schematic overview (Figure 4) has been added to summarize the genotype–phenotype relationships and illustrate the sequential logic used to construct the composite cardiovascular risk score, integrating genetic, bioinformatic, and clinical parameters. The diagram illustrates the study workflow from variant detection (LDLR, APOB, PCSK9) through in silico functional annotation (SIFT, PolyPhen-2, CADD, ΔΔG) and integration with clinical–biochemical parameters (LDL-C, TG/HDL ratio, BMI, xanthelasma) to derive the composite score for CVD risk stratification among FD patients.

While the composite risk score demonstrated promising diagnostic performance, its predictive strength has not yet been directly compared with conventional cardiovascular risk tools. Existing population-based scores such as Framingham or SCORE2 were not applied in the current analysis because they were derived from older, non-familial cohorts with substantially different baseline risk profiles [110–112]. Moreover, traditional algorithms underestimate risk in familial hypercholesterolemia because they omit genetic and zygosity information [104, 113, 114]. However, future work will involve direct benchmarking of the proposed composite score against Framingham, SCORE2, and familial-hypercholesterolemia–specific algorithms such as the Dutch Lipid Clinic Network (DLCN) score. This comparative validation, planned within the national Familial Dyslipidemia and Cardiovascular Disease Registry, will evaluate calibration, discrimination, and net reclassification improvement metrics [75, 115–117], thereby contextualizing the new model’s predictive utility.

### Implications for cardiovascular risk and genetic counseling

In summary, while *LDLR* mutations produce more extreme lipid levels and clinical signs than *APOB/PCSK9* mutations, all FD patients with severely elevated cholesterol are at risk for CVD, and the data show that no single gene group was statistically exempt from CVD. This reinforces that vigilant management of LDL-C is crucial in all genetically predisposed patients, and physical signs like xanthelasma indicate particularly high-risk individuals who warrant early and intensive therapy.

The genotype-phenotype insights have direct clinical relevance. Pathogenic*LDLR* variants, especially in the homozygous state, portend a greatly elevated and early-onset CVD risk, necessitating vigilant management and family screening *APOB* and *PCSK9* variants contribute to hypercholesterolemia as well, but their association with clinical CVD appears weaker. Recognizing these differences is important for personalizing risk assessment, for instance, an *APOB*-variant patient with moderate LDL elevation might have a slightly less ominous prognosis than a classic *LDLR* FH patient, though both warrant treatment. Finally, genetic findings in any FD patient should trigger cascade testing and thorough counseling, as per international guidelines, to mitigate cardiovascular risk through early identification and therapy in relatives [85]. This comprehensive approach; integrating genetic diagnosis, aggressive lipid management, and family screening is the cornerstone of contemporary FH care and is crucial to improving outcomes for these high-risk patients [74, 83].

### Public health and policy implications

From a public health standpoint, integrating genotype-based composite risk assessment into national screening frameworks could represent a cost-effective strategy to enhance early detection and prevention of familial dyslipidemia and related cardiovascular diseases. In settings such as Egypt and other middle-income countries, where universal genomic screening remains costly, a stepwise approach using biochemical markers, family history, and composite scoring can serve as a low-cost triage tool to identify high-risk individuals for confirmatory genetic testing [75, 118, 119]. This tiered strategy supports the principles of cascade testing allowing the identification of affected relatives through family tracing and could significantly reduce disease burden by enabling early lifestyle modification and pharmacologic intervention [74, 120]. Such precision-public-health approaches align with national cardiovascular-prevention programs and the World Health Organization’s targets for lipid management and premature NCD mortality reduction [16, 121].

From a policy perspective, these findings complement Egypt’s national Vision 2030 agenda and current health-system reforms aimed at reducing premature cardiovascular mortality. Integration of composite genomic–clinical risk assessment within the Universal Health Insurance (UHI) system and national NCD programs could enhance early identification and management of high-risk families. The approach aligns with the Ministry of Health and Population’s (MOHP) 2030 goals for precision prevention and equitable access to cardiovascular care [121–123]. Establishing a national familial dyslipidemia and cardiovascular disease registry, linked to genomic data, would also advance the Vision 2030 target of strengthening digital health infrastructure and population-based precision surveillance. Such integration of molecular diagnostics into routine screening pathways represents a practical step toward sustainable, prevention-oriented cardiovascular policy**.**

### Strengths and limitations

This study offers several important strengths. It is the first in Egypt to integrate clinical, biochemical, and genomic data with bioinformatics-based functional predictions to stratify CVD risk in adolescents and young adults with FD. The use of a targeted next-generation sequencing panel covering key genes (*LDLR, APOB*, and *PCSK9*) allowed for precise variant identification, while rigorous in silico tools (SIFT, PolyPhen-2, CADD, ΔΔG) enhanced variant interpretation. The development of a composite risk score incorporating clinical, genetic, and bioinformatic markers represents a novel and practical approach for personalized risk stratification. Additionally, the focus on a young, consanguineous population addresses a critical gap in the literature, with implications for early intervention in high-risk but understudied groups.

The present study has certain limitations that should be acknowledged. The relatively small sample size (n = 60), although supported by a formal power calculation demonstrating 80% power to detect group differences, may constrain external generalizability to broader populations and limit the detection of very rare variant associations. The cross-sectional design precludes causal inferences or assessment of temporal changes in cardiovascular outcomes. Moreover, the composite score was validated only within the study cohort and requires external validation in larger, multi-ethnic populations before broader clinical application. Functional validation of identified variants was not performed experimentally and relied on computational prediction, which, while informative, may not fully capture biological effects. Finally, limited access to longitudinal follow-up data restricts the ability to assess the prognostic utility of the composite score over time. Future multicenter and longitudinal studies are planned to extend these findings and externally validate the proposed model within diverse FD cohorts.

## Conclusions and recommendations

This study provided an integrated clinical, genetic, and computational assessment of FD in an Egyptian cohort, revealing distinct molecular and phenotypic profiles between patients with and without CVD. Triglyceride levels emerged as a key clinical discriminator of CVD risk, whereas LDL-C and HDL-C were less predictive. Genetic analysis showed a predominance of pathogenic *LDLR* variants among CVD cases, supported by bioinformatic predictions and protein stability models. In contrast, APOB and PCSK9 variants were less frequent and exhibited milder phenotypes.

The composite risk score developed; incorporating clinical, biochemical, and genetic data demonstrated high sensitivity and negative predictive value, especially among missense variant carriers, supporting its potential as a screening and risk-stratification tool. While highly sensitive in detecting CVD risk in genetically defined subgroups, its moderate specificity suggests that careful clinical interpretation is needed to avoid unnecessary interventions. For the broader FD population, the score offers a balanced, albeit less sensitive, approach. These findings highlight the importance of personalized risk assessment, guided by variant type and pathogenicity.

To enhance early detection and management, routine monitoring of triglyceride levels should be prioritized in FD care. Genetic screening, especially for pathogenic or likely pathogenic*LDLR*mutations should be integrated into clinical practice to enable early intervention. Functional characterization of variants of uncertain significance remains critical to improving diagnostic accuracy. The current composite risk score may benefit from further refinement through the inclusion of additional biomarkers to enhance specificity.

Furthermore, collaboration between clinical and genomic registries is strongly recommended to establish region-specific variant databases and population-specific risk thresholds. Building on this foundation, the authors plan to prospectively validate the composite risk score in larger and longitudinal cohorts through integration with the Egyptian Familial Dyslipidemia and Cardiovascular Disease Registry at the National Research Centre. This registry-based validation will allow evaluation of the score’s reproducibility, calibration, and prognostic value for cardiovascular outcomes across multiple governorates and potentially in regional EMRO collaborations, ensuring external generalizability and clinical applicability of the model.

Recent studies have demonstrated the diagnostic value of exome sequencing in rare Mendelian conditions and underscore the need for collaborative variant curation efforts in underrepresented populations [124]. Such efforts will enhance diagnostic accuracy and inform risk models tailored to genetically diverse and consanguineous populations.

Future studies should aim to validate the composite risk score in independent cohorts and assess its predictive utility through longitudinal follow-up. Incorporating this model into routine clinical practice could support genotype-guided therapy and personalized cardiovascular prevention strategies, particularly among high-risk youth in resource-limited settings.

## Supplementary Information

Supplementary Material 1

## Data Availability

The datasets used and/or analyzed during the current study are available from the corresponding author upon reasonable request.
