# Supplementary material for: Genomic and clinical predictors of cardiovascular disease in Familial dyslipidemia: risk stratification in Egyptian adolescents and young adults
Source: Lipids Health Dis. 2025 Dec 15;25:19. doi: 10.1186/s12944-025-02814-0 (PMC12821858; doi:10.1186/s12944-025-02814-0)
Supplement: Supplementary file 1 — Supplementary Material 1 [file 12944_2025_2814_MOESM1_ESM.docx]

**Table S1: All identified variants reported for all cases included in the present study.**

| **Gene** | **Transcript ID**  **(RefSeq)** | **cDNA Change**  **(HGVS)** | **Protein Change** | **Variant Type** | **dbSNP ID** | **Clinical Significance** |
| --- | --- | --- | --- | --- | --- | --- |
| **LDLR** | NM_000527.2 | c.501C>A | p.Cys167* | Nonsense | rs752596535 | Pathogenic |
|  |  | c.502G>A | p.Asp168Asn | Missense | rs200727689 | VUS |
|  |  | c.588del | p.Cys197Alafs*9 | Frameshift | Novel | Likely Pathogenic |
|  |  | c.907C>T | p.Arg303Trp | Missense | rs151207122 | Likely Pathogenic |
|  |  | c.1217G>A | p.Arg406Gln | Missense | rs552422789 | VUS |
|  |  | c.1255T>G | p.Tyr419Asp | Missense | rs879254847 | Likely Pathogenic |
|  |  | c.1301C>A | p.Thr434Lys | Missense | rs745343524 | Likely Pathogenic |
|  |  | c.1463T>C | p.Ile488Thr | Missense | rs879254913 | Pathogenic |
|  |  | c.1659C>G | p.Tyr553* | Nonsense | s564258872 | Pathogenic |
|  |  | c.1721G>A | p.Arg574His | Missense | re777188764 | Likely Pathogenic |
|  |  | c.1727A>C | p.Tyr576Ser | Missense | rs879254999 | Likely Pathogenic |
|  |  | c.1731G>T | p.Trp577Cys | Missense | rs875989928 | Likely Pathogenic |
|  |  | c.1757C>G | p.Ser586* | Nonsense | rs1555806455 | Pathogenic |
|  |  | c.1846-1G>A | P.? | Splice | rs879255051 | Pathogenic |
|  |  | c.1999T>C | p.Cys667Arg | Missense | rs150021927 | Pathogenic |
|  |  | c.2389G>A | p.Val797Met | Missense | rs750518671 | VUS |
|  |  | c.2416dup | p.Val806GlyfsTer11 | Frameshift | rs773618064 | Pathogenic |
|  |  | c.2552A>T | p.Gln851Arg | Missense | rs372328061 | Likely Pathogenic |
| **APOB** | NM_000384.2 | c.3740A>G | p.Tyr1247Cys | Missense | rs61741164 | VUS |
|  |  | c.10142T>A | p.Leu3381* | Nonsense | Novel | Likely Pathogenic |
|  |  | c.12137G>A | p.Arg4046Gln | Missense | rs149273387 | VUS / Pathogenic |
| **PCSK9** | NM_174936.4 | c.1487G>A | p.Arg496Gln | Missense | rs139669564 | VUS |

Abbreviation: HGVS: Human Genome Variation Society, RefSeq: Reference Sequence, dbSNP: Database of Single Nucleotide Polymorphisms (NCBI)

VUS: Variant of Uncertain Significance.

**Table S2: Functional Analysis of *LDLR, APOB, and PCSK9* Missense Variants**

| **Gene (Transcript ID)** | **cDNA Change (HGVS)** | **Protein Change** | **SIFT (Score)** | **PolyPhen-2 (Score)** | **CADD Score** | **ΔΔG (kcal/mol)** | **Reliability scoring index (RI)** | **Stability Interpretation** | **FD+CVD (n=30)** | **FD−CVD (n=30)** |
| --- | --- | --- | --- | --- | --- | --- | --- | --- | --- | --- |
| ***LDLR* (NM_000527.2)** | c.502G>A | p.Asp168Asn | Deleterious  (0.9) | Deleterious (1.00) | 25.8 | 0.62 | 1 | Decrease | 3 | 1 |
|  | c.907C>T | p.Arg303Trp | Deleterious  (0.00) | Deleterious (0.99) | 26.4 | -0.63 | 4 | Decrease | 6 | - |
|  | c.1217G>A | p.Arg406Gln | Uncertain (0.001) | Deleterious (0.99) | 35.0 | -0.89 | 8 | Decrease | 2 | - |
|  | c.1255T>G | p.Tyr419Asp | Deleterious (0.00) | Deleterious (1.00) | 25.5 | -0.75 | 5 | Decrease | 2 | - |
|  | c.1301C>A | p.Thr434Lys | Uncertain (0.014) | Uncertain (0.01) | 20.1 | -0.10 | 1 | Decrease | 3 | 2 |
|  | c.1463T>C | p.Ile488Thr | Deleterious (0.00) | Uncertain (0.001) | 26.5 | -4.22 | 9 | Decrease | - | 2 |
|  | c.1721G>A | p.Arg574His | Deleterious (0.00) | Deleterious (1.00) | 29.2 | -1.05 | 9 | Decrease | 1 | - |
|  | c.1727A>C | p.Tyr576Ser | Deleterious (0.00) | Deleterious (1.00) | 25.8 | -3.36 | 9 | Decrease | 1 | - |
|  | c.1731G>T | p.Trp577Cys | Deleterious (0.00) | Deleterious (1.00) | 29.5 | -0.66 | 3 | Decrease | 2 | - |
|  | c.1999T>C | p.Cys667Arg | Deleterious (0.00) | Deleterious (1.00) | 27.5 | -1.44 | 8 | Decrease | 5 | 2 |
|  | c.1999T>G | p.Cys667Gly | Deleterious (0.00) | Deleterious (1.00) | 31.0 | -4.32 | 10 | Decrease | 1 | 1 |
|  | c.2389G>A | p.Val797Met | Uncertain (0.006) | Uncertain (0.76) | 24.4 | -0.16 | 1 | Decrease | - | 1 |
|  | c.2552A>T | p.Gln851Leu | Uncertain (0.016) | Uncertain (0.011) | 23.3 | -1.62 | 7 | Decrease | - | 2 |
| ***APOB* (NM_000384.2)** | c.3740A>G | p.Tyr1247Cys | Uncertain (0.001) | Benign (0.00) | 22.0 | -1.06 | 1 | Destabilizing | - | 2 |
|  | c.12137G>A | p.Arg4046Gln | Benign (0.36) | Benign (0.00) | 13.9 | -0.89 | 3 | Destabilizing | 1 | - |
| ***PCSK9* (NM_174936.4)** | c.1487G>A | p.Arg496Gln | Benign (0.416) | Benign (0.02) | 15.3 | -1.84 | 8 | Destabilizing | 1 | - |

***Abbreviations:*** SIFT: Sort Intolerant from Tolerant, Polyphen-2: Polymorphism Phenotyping v2, CADD: Combined Annotation Dependent Depletion, ΔΔG: Change in Gibbs Free Energy (ΔG_mutant – ΔG_wild type; measures protein stability), FD: Familial Dyslipidemia, CVD: Cardiovascular Disease.
